# Modeling Within-Host Dynamics of Influenza Virus Infection Including Immune Responses

**DOI:** 10.1371/journal.pcbi.1002588

**Published:** 2012-06-28

**Authors:** Kasia A. Pawelek, Giao T. Huynh, Michelle Quinlivan, Ann Cullinane, Libin Rong, Alan S. Perelson

**Affiliations:** 1 Department of Mathematics and Statistics, Oakland University, Rochester, Michigan, United States of America; 2 Virology Unit, Irish Equine Centre, Johnstown, Naas, Co. Kildare, Ireland; 3 Theoretical Biology and Biophysics, Los Alamos National Laboratory, Los Alamos, New Mexico, United States of America; Emory University, United States of America

## Abstract

Influenza virus infection remains a public health problem worldwide. The mechanisms underlying viral control during an uncomplicated influenza virus infection are not fully understood. Here, we developed a mathematical model including both innate and adaptive immune responses to study the within-host dynamics of equine influenza virus infection in horses. By comparing modeling predictions with both interferon and viral kinetic data, we examined the relative roles of target cell availability, and innate and adaptive immune responses in controlling the virus. Our results show that the rapid and substantial viral decline (about 2 to 4 logs within 1 day) after the peak can be explained by the killing of infected cells mediated by interferon activated cells, such as natural killer cells, during the innate immune response. After the viral load declines to a lower level, the loss of interferon-induced antiviral effect and an increased availability of target cells due to loss of the antiviral state can explain the observed short phase of viral plateau in which the viral level remains unchanged or even experiences a minor second peak in some animals. An adaptive immune response is needed in our model to explain the eventual viral clearance. This study provides a quantitative understanding of the biological factors that can explain the viral and interferon kinetics during a typical influenza virus infection.

## Author Summary

Influenza, commonly referred to as the flu, is a contagious respiratory illness caused by influenza virus infections. Although most infected subjects with intact immune systems are able to clear the virus without developing serious flu complications, the mechanisms underlying viral control are not fully understood. In this paper, we address this question by developing mathematical models that include both innate and adaptive immune responses, and fitting them to experimental data from horses infected with equine influenza virus. We find that the innate immune response, such as natural killer cell-mediated infected cell killing and interferon's antiviral effect, can explain the first rapid viral decline and subsequent second peak viremia, and that the adaptive immune response is needed to eventually clear the virus. This study improves our understanding of influenza virus dynamics and may provide more information for future research in influenza pathogenesis, treatment, and vaccination.

## Introduction

Despite vaccines and antiviral agents, influenza A virus infection remains a major public health problem worldwide. Seasonal and pandemic influenza results in approximately 3 to 5 million cases of severe illness and approximately 250,000 to 500,000 deaths worldwide [1]. Influenza viruses primarily infect and replicate in epithelial cells [2]. The immune response to influenza virus infection plays an important role in controlling the virus within a host. The nonspecific innate immune response provides the first line of defense, which reacts immediately upon infection and involves generating a variety of chemotactic, proinflammatory and antiviral cytokines [3]. An important cytokine produced during the innate immune response is type I interferon (mainly IFN-α/β). IFN-α/β has been shown to stimulate resistance to infection in the neighboring cells by inducing the expression of many IFN-stimulated gene products, including antiviral proteins, such as protein kinase R, PKR [4]. Depletion of key IFN signaling proteins in mice results in greater mortality, accompanied by systemic (as opposed to respiratory-restricted) infection [5]. In addition, IFN is able to activate immune system cells, such as natural killer (NK) cells, during the early stage of infection, which can destroy infected cells [6]–[10]. The secretion of IFN-α/β by infected epithelial cells is also important for the initiation of the antigen-specific adaptive immune response [11], [12], which in mice takes approximately 5 days to begin in the lung [13]. The adaptive immune response mainly consists of cytotoxic CD8^+^ T cells eliminating infected cells and antibodies neutralizing the virus [11]. It is important for clearing the virus and provides immunity against future influenza virus infections. Because of limited information about influenza pathogenesis and the host immune response in humans, various animal models, such as mice, ferrets, and horses [14]–[17], have been used to obtain a better understanding of the biological mechanisms underlying viral control.

A number of mathematical models have been developed to study the dynamics of influenza virus infection and immune responses [13], [18]–[28] (also see recent reviews in [29]–[31]). By fitting a simple viral dynamic model to the data derived from 6 experimentally infected human volunteers, Baccam et al. [20] showed that target cell limitation can explain the kinetics of influenza A virus infection in humans. Both innate [18], [20], [28] and adaptive immune responses [21], [22], [24] have also been incorporated into the basic model to evaluate the effect of immune responses on viral control. In a recent study, Miao et al. [13] quantitatively investigated the innate and adaptive immune responses to primary influenza A virus infection in mice. They compared the half-life of infected epithelial cells and free virus before and during a virus-specific immune response (about 5 days post-infection). Lee et al. [27] developed a two-compartment model to study the contributions of different factors, such as antigen presentation and activation of naive T and B cells, CD4^+^ T cell help, CD8^+^ mediated cytotoxicity, and antibody, to the control of influenza A virus infection. These studies provide a quantitative understanding of the host immune response in controlling virus replication.

The relative contributions of target cell availability and immune responses to viral control remain unclear. In a recent study, Saenz et al. [19] estimated the numbers of viral-antigen-positive cells in the lungs of ponies at days 2.5, 4.5, and 5.5 after challenge with equine influenza virus (EIV). The result indicated that up to 5% of bronchiole cells were infected at any one time, yielding an estimated total cell loss of about 27% by the end of the infection. This suggests mechanisms for viral control in addition to target cell depletion [20], and motivates the development of a model that includes a strong innate immune response to explain the clearance of virus during infection [19]. However, the model in [19] is unable to capture a number of important features of the viral kinetics observed in 6 ponies, e.g., the viral peak in most of the ponies, the rapid and substantial viral decline after the peak (2 to 4 log decline within 1 day), and a short plateau phase in which the viral load remained unchanged or even experienced a minor second peak in some ponies [19]. In this study, we develop mathematical models based on several possible biological mechanisms that attempt to explain all of these observations. Our objective is to investigate which biological parameters can give rise to the viral load change observed during an uncomplicated influenza virus infection.

## Materials and Methods

### Experimental data

The data we studied were from an experimental challenge of 6 unvaccinated ponies infected with EIV A/eq/Kildare/89 (H3N8) [16]. Nasal secretions (NS) were collected daily for 10 days post-challenge and number of copies of influenza virus RNA per milliliter (ml) was quantified. Blood samples were also collected to quantify the fold changes in cytokine expression including IFN for days 1 through 5 post-challenge compared to the day prior to challenge. We used both the viral load and the IFN fold change data in this study. High antibody titers were detected by the single radial haemolysis (SRH) assay 14 days post-challenge in the horses.

Upon infection, the viral load increased rapidly and reached its peak at day 2 for all ponies. There was a wide variation in the peak level. The highest was approximately 10^8^ copies of viral RNA/ml of NS (pony 2), while the lowest was 10^4^ copies/ml of NS (pony 6). After the peak, the viral load experienced a rapid and substantial decline (about 2 to 4 logs within 1 day). All the ponies had a viral plateau and some experienced a minor but obvious second peak. After the viral plateau/second peak, there was a second viral decline starting around day 6. In 4 out of the 6 ponies, the viral load decreased to below the detection limit by day 8. The rest of the ponies had undetectable viral load at day 9. During the infection, IFN expression increased substantially reaching a peak on day 2 in 5 of the 6 ponies, followed by a rapid decrease to the pre-infection level [16], [19]. The peak of IFN-fold change ranged from approximately 1 (pony 3) to more than 10 (pony 6).

### Mathematical model

We developed a model to study the within-host dynamics of EIV infection in horses. It is described by the following system of equations
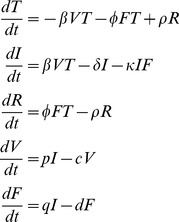
(1)The model has five variables: target cells (*T*), productively infected cells (*I*), uninfected cells that are refractory to infections (*R*) because of IFN-induced antiviral effect [32], free virus (*V*), and IFN (*F*). The term *βVT* represents the rate of infection when virus encounters susceptible target cells. IFN induces an antiviral effect and enables uninfected cells to become refractory to infection at rate 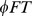. Cells in the refractory state revert back to the susceptible state at rate *ρ*. Infected cells are assumed to die at per capita rate *δ*.

Prior to the emergence of the antigen-specific adaptive immune response, we assume *δ* is a constant *δ_I_*. This rate (*δ*) becomes *δ_A_ = δ_m_-(δ_m_-δ_I_)e^−σ(t-μ)^* after the adaptive immune response emerges, where *μ* is the time at which the adaptive immune response emerges, *δ_m_* is the maximum death rate of infected cells in the presence of an adaptive immune response, and *σ* determines how fast the death rate increases from *δ_I_* to the saturation rate *δ_m_*. Because we only model the dynamics for a few days after the adaptive immune response emerges, we modify the time-varying death rate to *δ_A_* = *δ_I_e^σ(t-μ)^* without using the maximum constant *δ_m_*. In this way, the number of parameters introduced is reduced by 1. Another method that explicitly includes the adaptive immune response as an additional variable in the model was also examined and the results are mentioned in the Discussion section.

**Figure 1:**
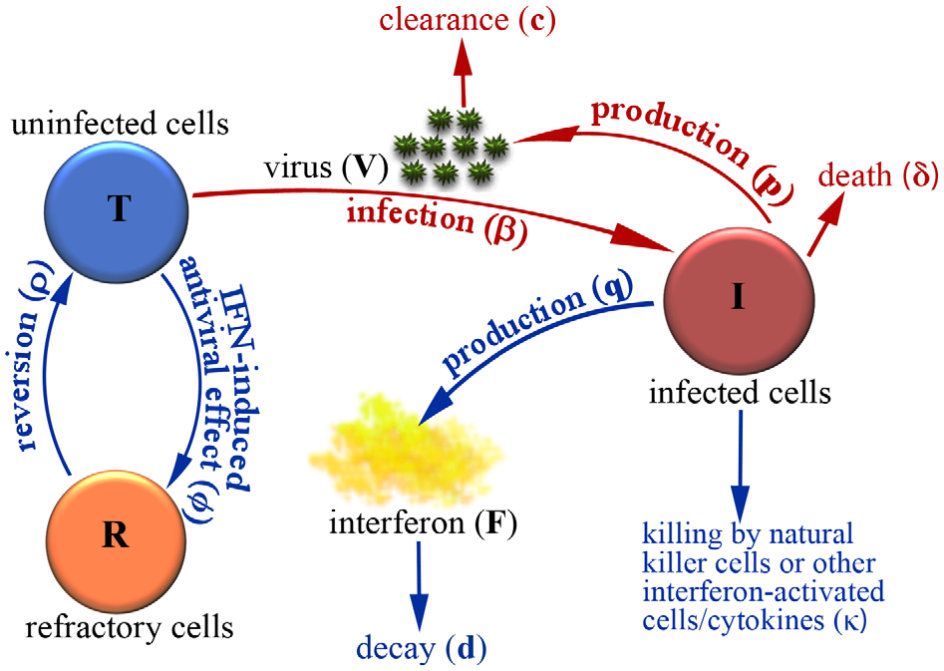
Schematic representation of Eq. (1).

In the early stage of influenza virus infection, NK cells can be activated by IFN to induce cytolysis of infected epithelial cells and play an important role in the innate immune response [6], [7], [8], [9], [10]. Here, we assume the number of activated NK cells is proportional to the level of IFN and use the mass action term 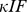 to represent the killing by NK cells. Note that killing by NK cells is an important, but not the only factor leading to the loss of infected cells. Cytokines or proteins released by other cells such as macrophages [33] during the innate immune response can also promote increased lung epithelial apoptosis following influenza virus infection [34], [35]. Infected cells are assumed to produce virus at rate *p* and free virus is cleared at rate *c* per virion. As in the previous models by Baccam et al. [20] and Saenz et al. [19], loss of virions due to infection has been neglected. Since an infected cell may produce as many as 20,000 virions [36], the loss of one virion to produce an infected cell can be neglected. IFN is secreted by infected cells at rate *q* and decays at rate *d*. A schematic diagram of Eq. (1) is shown in Figure 1. Variables and parameters are summarized in Table 1.

**Table 1:** Variables, parameters, and values used in Eq. (1).

Symbol	Definition	Unit	Value
*_T_*	Uninfected epithelial cells that are susceptible to infection	cells	initial value: 3.5×10^11^ [39]
*_I_*	Infected epithelial cells	cells	initial value: 0
*_R_*	Epithelial cells in the refractory state	cells	initial value: 0
*_V_*	Viral load	RNA copies (ml NS)^−1^	initial value: fitted
*_F_*	Interferon	IFN fold change	initial value: 1
*_β_*	Infection rate	(RNA copy)^−1^ ml NS day^−1^	fitted
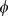	IFN-induced antiviral efficacy	(IFN fold change)^−1^ day^−1^	fitted
*_ρ_*	Reversion rate from refractory	day^−1^	fitted
*_δI_*	Death rate of infected cells before the adaptive immune response emerges	day^−1^	2 [31], [37]
*_δA_*	Time-varying death rate of infected cells during the adaptive immune response	day^−1^	see text
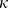	Killing rate of infected cells by NK cells	(IFN fold change)^−1^ day^−1^	fitted
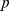	Viral production rate	RNA copies (ml NS)^−1^ day^−1^ cell^−1^	fitted
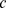	Clearance rate of free virions	day^−1^	fitted
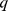	Production rate of IFN	IFN fold change day^−1^ cell^−1^	fitted
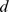	Decay rate of IFN	day^−1^	fitted

### Parameter values and data fitting

We fixed some parameters and estimated the rest by fitting the model to both the viral load and IFN data. The lifespan of infected cells prior to the emergence of the adaptive immune response, 1/*δ_I_*, was fixed to 0.5 days [31], [37], which is the value used in previous modeling studies [19], [21]. Because no CD8^+^ T cell data were obtained in this experiment, we chose the time at which the adaptive immune response emerges (*μ*) according to the second viral decline. For example, we chose *μ* = 7 days for pony 1 and *μ* = 6 days for pony 2. A similar method has been used previously in analyzing acute HCV infection kinetics in chimpanzees [38]. We also included a delayed adaptive immune response explicitly in the model and obtained similar results (see Discussion). The initial population of epithelial cells in the equine respiratory tract was fixed at *T_0_ = *3.5×10^11^ cells [39]. We assume all such cells are target cells, as used in Saenz et al. [19], although H3N8 viruses prefer to infect α 2,3 sialic acid glycan-expressing cells [40] and thus the number of target cells could be less than assumed here. We include sensitivity test to a number of parameters including the initial number of target cells below. We set the initial population of infected cells and refractory cells to 0, and the initial IFN fold change to 1, i.e., no change, as given in the data set. The remaining parameters were estimated from data fitting. Note that some parameters, such as the infection rate constant *β* and the viral production rate *p*, do not have physiological values because they are in the unit of ml of nasal secretions.

We fit the model to both the viral load and IFN data of each pony using the commercial software package Berkeley Madonna (Version 8.3.18). The obtained parameter values were based on the best nonlinear least squares fit of the model equations to the data set, i.e., the program minimized the root mean square (RMS) between data points and the corresponding model predictions, given by
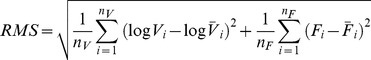
(2)where the number of viral load and IFN fold change measurements for an individual pony are denoted by *n_V_* and *n_F_*, respectively. Viral load data is given by 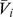 and the analogous value given by our model is *V_i_*. Similarly, the measured IFN fold change is 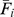 and the corresponding model prediction is *F_i_*. The first data point below the detection limit (100 copies/ml of NS) was assumed to be 1 copy/ml of NS. Other values, such as half of the detection limit, can also be used [41], which will affect the estimate of the parameter *σ* in this study. There are also other approaches to incorporating left-censored measurements [42]. We did not include the viral load data under the detection limit after the first undetectable data point. Equal weights for both viral titer and IFN data were employed because they are approximately in the same range. Using different weights or normalized data (each value is divided by the maximum) generates a similar fit, although the estimates of parameter values can be different.

### Approximation of viral decline after the peak using the target cell limited model

The target cell limited model was used in [20] and described by the following equations: *dT/dt = −βVT*, *dI/dt = βVT-δI*, and *dV/dt = pI-cV*. Assuming *t_peak_* is the time at which the viral load achieves its peak, we have *pI = cV* at *t = t_peak_*. Thus, *I(t_peak_) = cV(t_peak_)/p*. Because target cells are nearly depleted around the peak of infection in this model [20], we assumed *T≈0* for a short time period after *t_peak_*, and solved for *I(t)*. This assumption was also used in [23] to obtain an approximation for the decay after the peak using the model with an eclipse phase. The solution is 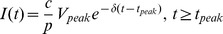. Substituting this into the *V(t)* equation and solving for *V(t)*, we have 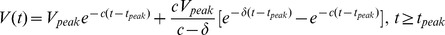. Thus, the predicted viral load reduction 1 day after the peak is 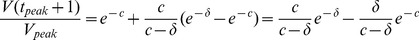. As *c* is typically much larger than *δ* (Table 2), this ratio is mainly determined by the value of *δ* . For *δ* in the range of (0, 4.5) day^−1^, which covers most of the estimates in the literature [20], the ratio is always greater than 0.01 for any positive value of *c*. This implies that for any value of *δ*<4.5 day^−1^, the target cell limited model generates <2 log decline within 1 day after the peak. The actual viral load reduction predicted by the model should be less than this approximation because we assumed *T*≈0 over the interval [*t_peak_*, *t_peak_*+1]. Numerical results show that to obtain a 3 log decline within 1 day after the peak, *c* should be >12 day^−1^ and *δ* needs to be >8 day^−1^. To attain a 4 log decline, *c* should be >18 day^−1^ and *δ* needs to be >10 day^−1^.

**Table 2:** Parameter values of the best fits of Eq. (1) to experimental data.

Pony	*<*	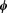	*ρ*	*κ*	*p*	*c*	*q*	*d*	*σ*
	(RNA copy)^−1^ ml NS day^−1^	(IFN fold change)^−1^ day^−1^	day^−1^	(IFN fold change)^−1^ day^−1^	RNA copies (ml NS)^−1^ day^−1^ cell^−1^	day^−1^	IFN fold change day^−1^ cell^−1^	day^−1^	
1	8.3×10^−6^	6.9×10^−2^	1.0×10^−2^	1.6	7.7×10^−5^	20	6.1×10^−10^	0.85	1.0
2	1.1×10^−7^	2.2×10^−2^	1.0×10^1^	5.7	2.4×10^−2^	14	4.5×10^−10^	2.2	0.37
3	8.5×10^−5^	1.2×10^0^	6.7×10^−2^	11	1.6×10^−5^	9.8	3.3×10^−10^	2.0	0.92
4	1.2×10^−6^	1.1×10^−1^	5.1×10^0^	1.2	9.6×10^−4^	19	3.8×10^−10^	1.9	0.23
5	2.8×10^−7^	5.3×10^−1^	3.7×10^−2^	3.2	6.8×10^−3^	20	1.9×10^−9^	1.9	1.2
6	1.9×10^−4^	2.5×10^−2^	2.0×10^−1^	2.4	8.1×10^−6^	5.8	2.1×10^−9^	2.4	2.2
Average	4.7×10^−5^	3.3×10^−1^	2.6×10^0^	4.2	5.3×10^−3^	15	9.6×10^−10^	1.9	0.99

### Statistical analysis

To statistically compare the best fits using model 1 (Eq. (1)) and model 2 (setting κ to 0 in model 1, i.e., no killing of infected cells by NK cells), we performed an *F*-test. An *F*-test is used to compare two nested models used to fit the same data set to determine whether the model with more parameters statistically improves the fit. The improvement is considered to be statistically significant if the *p-*value is less than 0.05. We begin with the calculation of the *F-*value as follows:
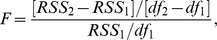
where *RSS* is the sum of squared residuals between model predictions and data. The *RMS* value generated from Berkeley Madonna is the root of the mean squared residuals. Hence, *RSS = n•(RMS)^2^*, where *n* is the number of data points. The subscripts 1 and 2 represent model 1 and model 2, respectively. The degree of freedom associated with *RSS* is *df = n-m*, where *m* is the number of fitted parameters. Note that *μ*, the time at which the adaptive immune response emerges, was counted as a fitted parameter although we fixed it according to the second viral decline. To compute the *p*-value, we calculated the *F* distribution evaluated at the *F-*value with (*df_2_-df_1_*, *df_1_*) degrees of freedom. Comparison between models was performed individually for all the ponies.

## Results

### Overview of the best fits of Eq. (1) to experimental data

**Figure 2:**
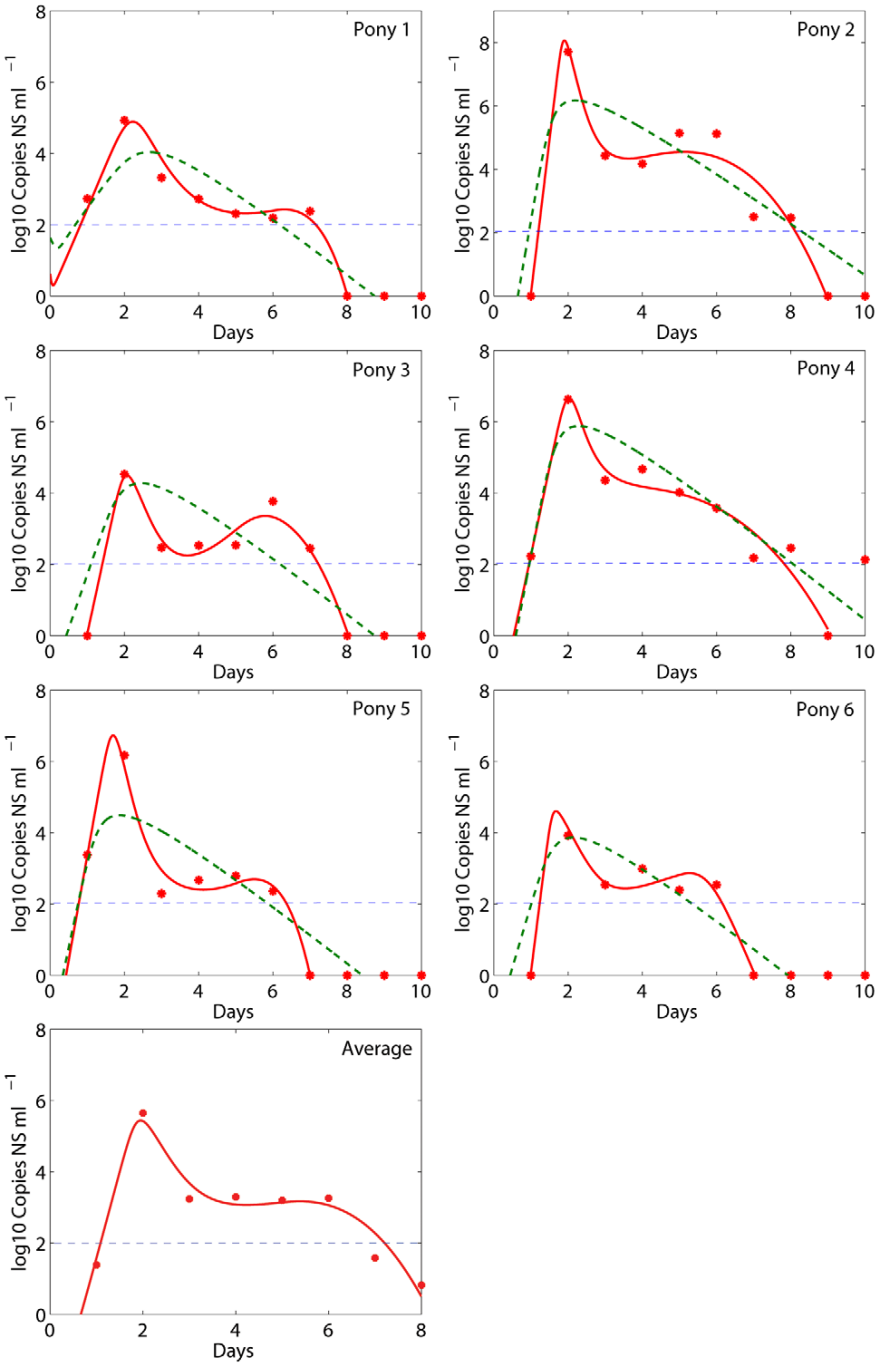
Model comparisons with viral load data. Best fits of Eq. (1) (solid red) and the Saenz et al. model (dashed green) to the viral load data (filled red circles) were shown. The horizontal dashed blue line represents the detection limit of the viral titer, i.e., 100 RNA copies per ml of nasal secretions. Data below the detection limit were plotted as 1 RNA copy per ml of nasal secretions.

**Figure 3:**
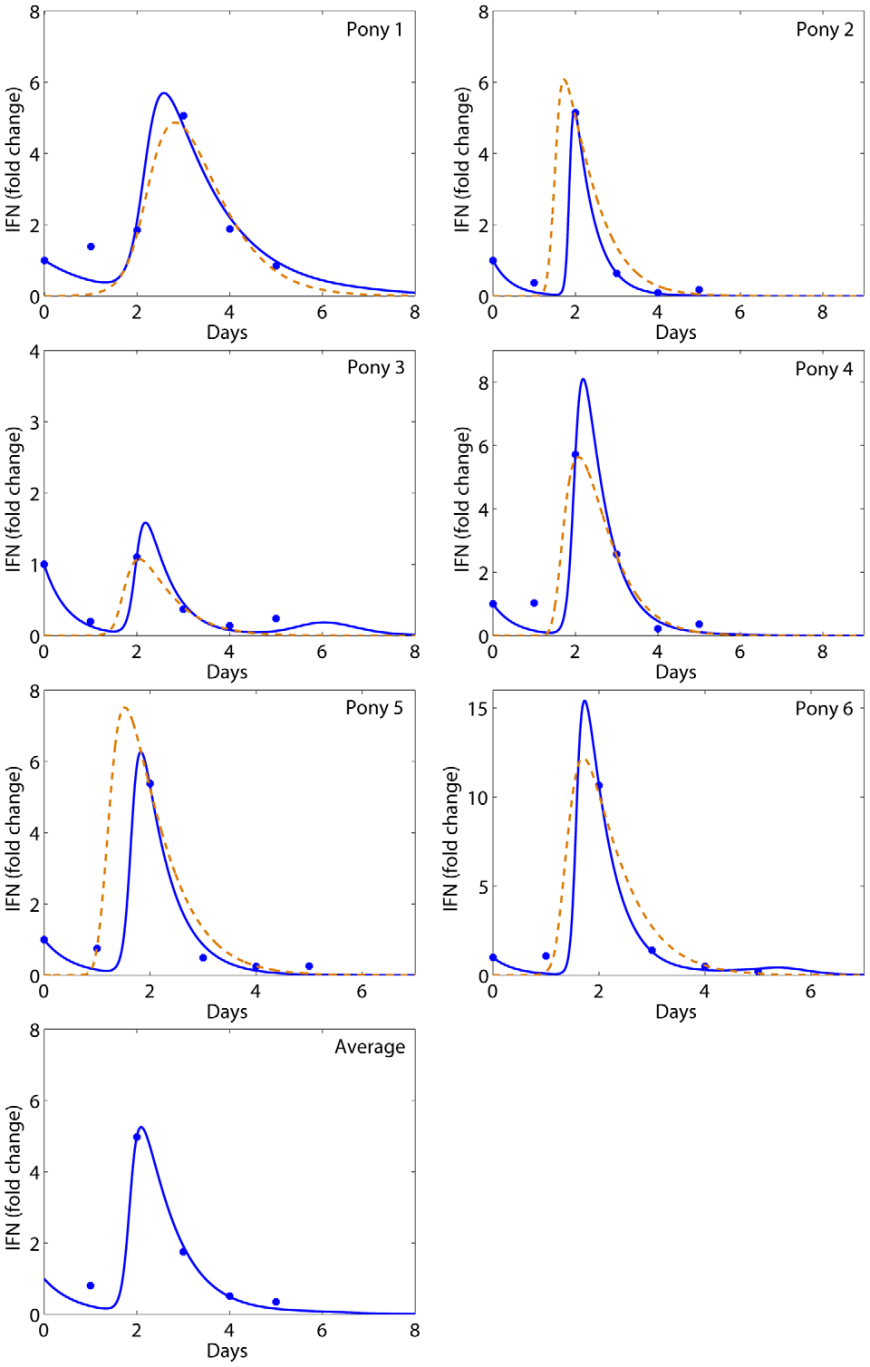
Model comparisons with IFN data. Best fits of Eq. (1) (solid blue) and the Saenz et al. model (dashed orange) to the IFN fold change data (filled blue circles) were shown.

We fit the predicted values of *V(t)* and *F(t)* in Eq. (1) to the viral load and IFN (fold change) kinetic data, respectively, of each pony. The best fits, shown in Figures 2 (red solid) and 3 (blue solid), indicate that Eq. (1) agrees with both the viral load and IFN data well. Parameter values corresponding to the best fits are given in Table 2. Note that the estimates of some parameters, such as the infection rate *β* and the viral production rate *p*, have large variations. This is expected because there is a large variation (up to 4 logs) in the peak viral load of the 6 ponies. We also fit the model to the average data of the 6 ponies (Figures 2 and 3). The average data show similar kinetic changes of viral titer and IFN, and the best-fit model agrees well with the data.

For comparison, we also plotted the best fits (dashed lines in Figures 2 and 3) of the Saenz et al. model [19] to the same viral load and IFN data. Our model improves the viral load data fits in several aspects. First, our fits capture the viral peak in all 6 ponies. Second, the fits achieve the rapid and substantial viral decline within 1 day after the peak in all ponies. Third, the fits generate a period of viral plateau and/or a second peak. Lastly, our fits generate the rapid second viral decline to below the detection limit in all 6 ponies. Detailed explanations and possible biological mechanisms for these viral load changes are given below.

### Rapid and substantial viral decline after the peak

The viral loads in all 6 ponies experienced a 2 to 4 log decline within 1 day after the peak [16], [19]. Similar viral declines were also observed in 6 volunteers experimentally infected with influenza A virus [20]. What causes such a rapid and substantial viral decline within a short period of time? The data fits using both the target cell limited model in [20] and the modified model in [19] did not capture this feature. In fact, using the target cell limited model we can derive an approximation of the viral load reduction 1 day after the peak (see Materials and Methods). For most of the estimates of the infected cell death rate in the literature, the target cell limited model cannot generate a >2 log decline within 1 day after the viral peak. This suggests that other factors not included in the target cell limited model may be responsible for this dramatic viral decline. We tested different models based on several possible biological mechanisms (see below) and found that the model shown in Eq. (1) can reproduce the viral load change observed in the 6 ponies. The rapid viral decline after the peak is mainly due to the combination of two factors: the decline of target cells because of their conversion to the refractory class (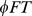 in Eq. 1) by IFN's antiviral effect, and the killing of infected epithelial cells (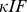 in Eq. 1), possibly mediated by IFN activated NK cells during the innate immune response.

**Figure 4:**
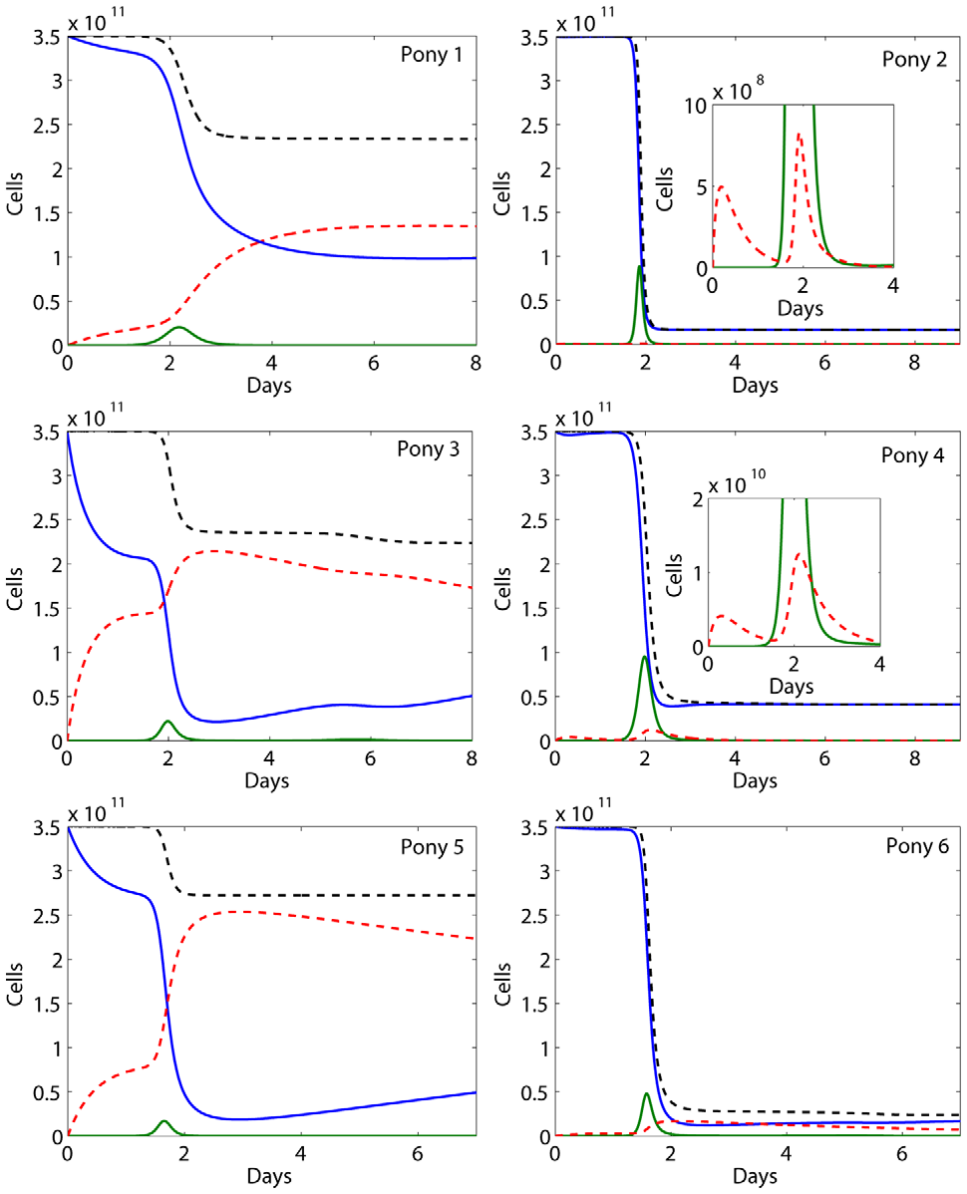
The changes of cell populations predicted by **Eq. (1)** based on the best fits. Solid blue represents susceptible cells, solid green represents infected cells, dashed red represents cells in the refractory state, and dotted black represents the total number of cells. The curves were zoomed in for ponies 2 and 4 to show the level of refractory cells.

We plotted the changes of uninfected target cells (solid blue), infected cells (solid green), refractory cells (dashed red), and total cells (dotted black) in Figure 4. The number or percentage of infected epithelial cells is low compared to the prediction of the target cell limited model [20]. In contrast with the predictions of the Saenz et al. model [19], the level of uninfected target cells remains high (>10^10^ cells) for all the ponies during the entire infection course. The reversion of cells from the refractory to the susceptible class (*ρR*) prevents uninfected target cells from decreasing to a very low level. This suggests that in addition to target cell depletion, cytolysis of infected cells mediated by IFN activated cells such as NK cells during the innate immune response may be responsible for the viral decline during the early stage of influenza virus infection.

**Figure 5:**
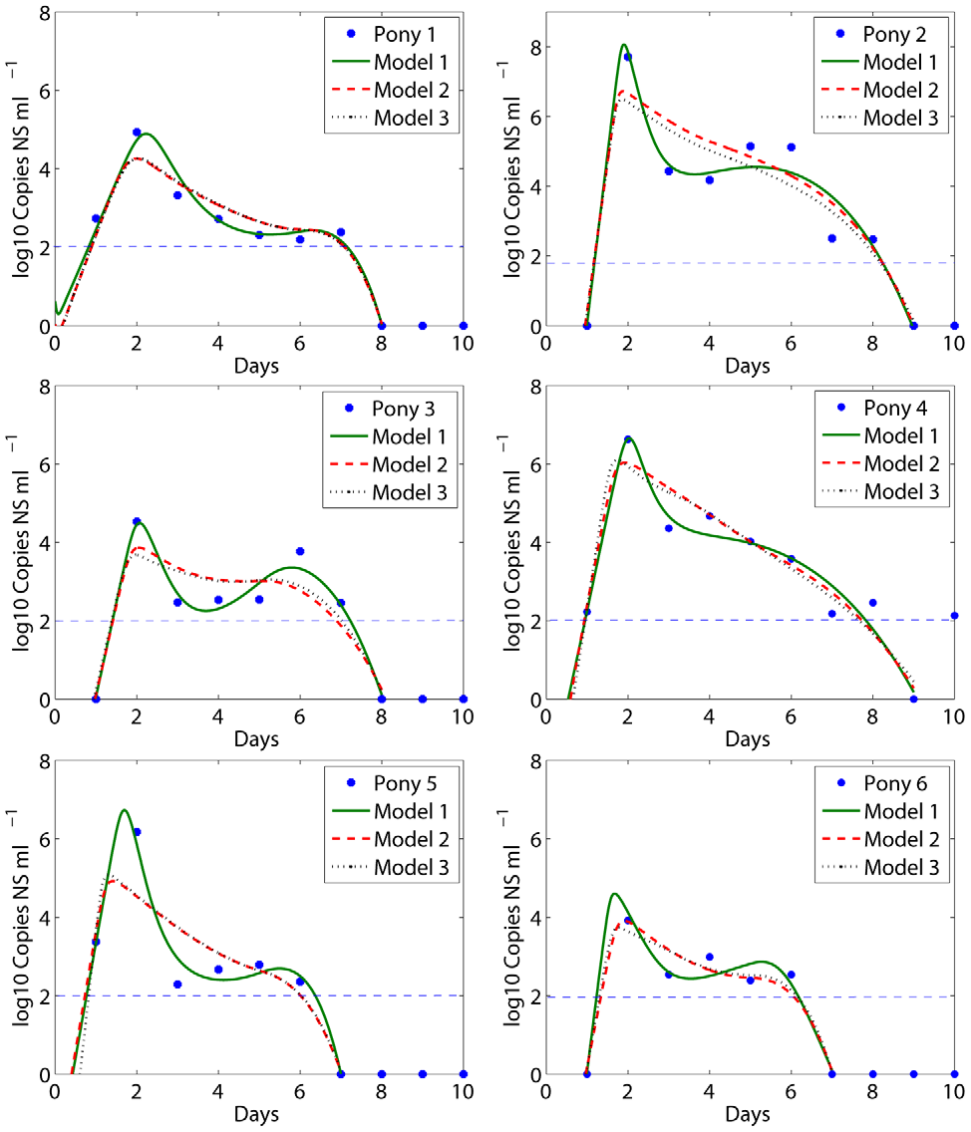
Best fits of different models to experimental data. Model 1 is described by Eq. (1). Model 2 is Eq. (1) with κ = 0, i.e., no killing of infected cells by NK cells. Model 3 is model 2 assuming the viral production rate is 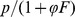. The detection limit of the viral titer is 100 RNA copies per ml of nasal secretions. Data below the detection limit were plotted as 1 RNA copy per ml of nasal secretions.

To further test if a model that only includes the refractory class without NK cell-mediated infected cell killing (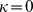 in Eq. 1; referred to as model 2) can explain the first rapid viral decline, we fit model 2 to the same experimental data (dashed lines in Figure 5 for viral load and Supporting Figure S1 for IFN fold change). We found model 2 cannot generate the rapid viral load decline after the peak. We also tested a model assuming that IFN only reduces the viral production rate (i.e., assuming 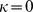 and replacing *p* with 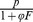 in Eq. (1); this is referred to as model 3) and found this model could not generate the first rapid viral decline either and yielded dynamics very similar to model 2 (dotted lines in Figure 5). Thus, the cell-mediated lysis of infected cells during the innate immune response plays a critical role in generating the first rapid viral decline in our model. We calculated the error between modeling predictions and experimental data (RMS) for different models. The RMS values are given in Table 3. Model 1 generated the smallest error for each pony.

**Table 3:** Comparisons of the best fits using different models.

Pony	RMS of model 1*	RMS of model 2*	Number of data points**	*p-*value for *F*-test	RMS of Saenz et al. model***
1	0.695	0.951	14	0.181	1.442
2	0.651	1.061	15	<0.05	1.873
3	0.320	0.718	14	<0.05	1.613
4	0.768	0.953	15	0.202	1.352
5	0.618	1.027	13	0.157	1.610
6	0.695	0.845	13	0.400	1.802

*: Model 1 is described by Eq. (1). Model 2 is Eq. (1) with κ = 0, i.e., there is no killing of infected cells by NK cells.

**: We did not include data points of viral titer under the detection limit after the first undetectable data point.

***: The RMS value was calculated by Eq. (2) in the Materials and Methods. These values are different from those presented in Saenz et al. [19] because the percentage of infected cells is not included (see text).

We compared the best fits of using model 1 and model 2 by performing an *F*-test , which determines which one of the two nested models provides a better data fit from a statistical standpoint (Materials and Methods). The results given in Table 3 show that model 1 provides significantly better fits for ponies 2 and 3 (with the *p*-value<0.05). For the other ponies, the *F*-test shows that there is a statistical trend supporting model 1 (with the *p*-value from 0.1 to 0.4). We also compared the best fits using the modified Akaike Information Criterion (AICc) (Supporting Text S1). Model 1 is supported over model 2 for each pony (Table S4).

We did not statistically compare the fits of model 1 with the Saenz et al. fits [19] because the objective functions minimized during data fitting are different. Saenz et al. [19] incorporated the percentage of infected cells in their fitting. We did not include this because the data of the percentage of infected cells were from a different study [43]. The errors listed in Table 3 and the fitted curves (Figures 2 and 3) show that our fits improve those using the Saenz et al. model.

### Viral plateau and second peak

The phenomenon of bimodal viral titer peaks in most ponies [16] was also observed in other studies with influenza virus infection [44], [45], [46]. The target cell limited model [20] and the Saenz et al. model [19] cannot generate bimodal virus titer peaks. Adding the effect of IFN and a time delay in its production into the target cell limited model was shown to be able to generate bimodal peaks [20]. However, the fits obtained by Baccam et al. [20] using this model did not agree well with the data. Our fits using model 1 generated an obvious bimodal behavior (Figure 2). The level of IFN peaked around day 2 and then declined rapidly (Figure 3), concordant with the emergence of viral plateau/second peak (Figure 2). Thus, the viral plateau and the second viral titer peak can be explained by the loss of the IFN-induced antiviral effect (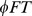 in Eq. 1). Increased availability of susceptible cells due to reversion from the refractory state (*ρR* in Eq. 1) can also contribute to the viral plateau/second peak. From our data fits we estimated that the rate (ρ) at which refractory cells (*R*) revert from the refractory to the susceptible state is on average 2.6 per day. The reversion rate is also important in preventing uninfected target cells from decreasing to a very low level. Sensitivity tests of the model predictions to a number of parameters, including 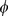 and *ρ*, are given below.

### Sensitivity test

**Figure 6:**
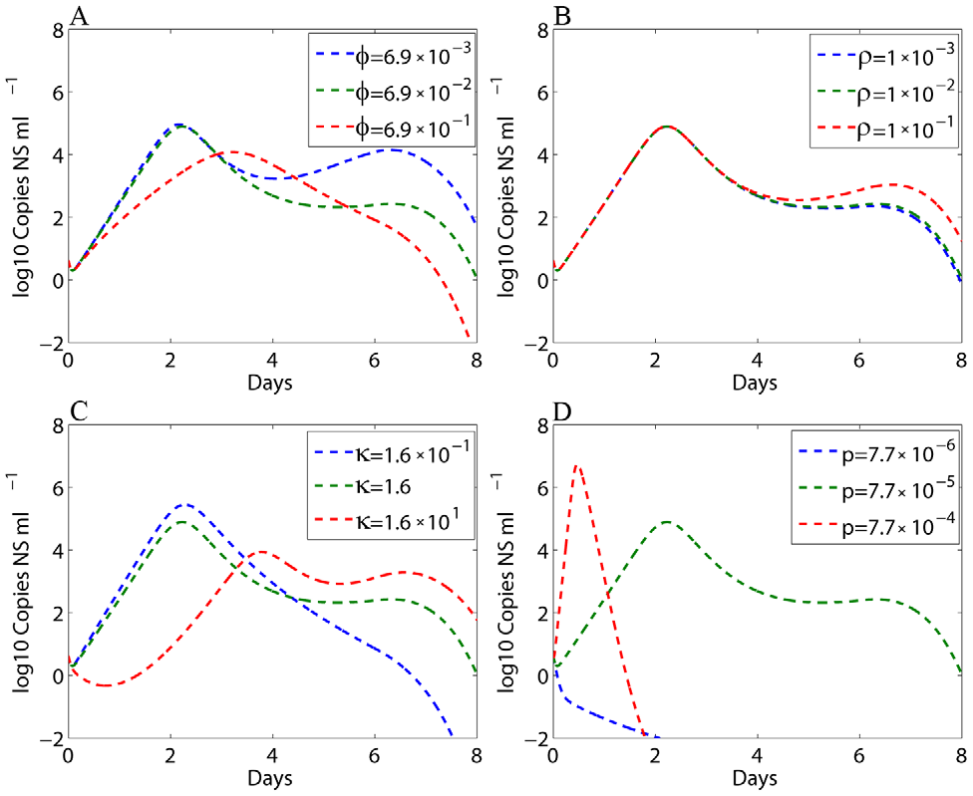
Sensitivity tests of the predicted viral load of pony 1 to model parameters. The parameter in the legend was varied (10-fold larger or smaller than the estimate in Table 2) while the remaining parameters were fixed and chosen from Table 2.

We examined the sensitivity of the predicted viral load of pony 1 to several parameters, including 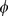, *ρ*, *κ*, and *p* (Figure 6). More sensitivity tests of the predicted viral load and IFN to other parameters and contour plots are presented in Supporting Figures S2, S3, S4, S5, S6, S7, S8. Sensitivity tests show that the IFN's antiviral efficiency (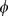) and the reversion rate (*ρ*) are important in generating the viral plateau and the second peak (Figure 6A, B). A large value of 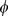 can also yield a rapid first viral decline. However, this will eliminate the viral plateau and the second peak (Figure 6A). Increasing the infected cell killing rate constant 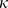 alone will decrease the first viral peak and increase the second peak (Figure 6C). A large value of the viral production rate *p* (Figure 6D) or the infection rate *β* (Figure S2) can achieve the first viral peak. However, they will significantly reduce the time for the viral titer to reach the peak. These sensitivity tests suggest that the cell-mediated lysis of infected cells (*κ*) and the IFN's antiviral effect (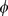) during the innate immune response are the major factors responsible for the first rapid viral decline and subsequent viral plateau/second peak.

Since the initial number of target cells of H3N8 virus infection could be less than 3.5×10^11^ cells (*T_0_*), the estimate of total epithelial cells in the equine respiratory tract [39], we reduced it from *T_0_* to 75% or 50% of *T_0_*. The simulation in which the other parameters are assumed to be unchanged shows that a small initial number of target cells can delay the time to reach the first viral peak, reduce the magnitude of the peak viremia, and eliminate the viral plateau (Figure S2). However, data fitting using 75% and 50% of *T_0_* still generates good fits to the experimental data (see Figure S2 for the fit to the viral load data of pony 1).

## Discussion

The biological factors responsible for viral control during influenza virus infection remain unclear. Earlier work [20] suggested that the viral decline after the peak could be explained by a limitation in the availability of target cells. However, a recent study by Saenz et al. [19] estimated that <5% of epithelial cells are infected at any one time and that the total epithelial cell loss is <30% by the end of the infection. They modified the target cell limited model by including an IFN-induced antiviral state of uninfected cells [19]. However, their modified model is still essentially a target cell limited model — uninfected target cells move to the refractory class, causing the depletion of susceptible cells and hence the viral titer declines after reaching the peak. Numerical simulations also confirmed this prediction (Figure 3 in [19]). As we analytically showed in Materials and Methods, the target cell limited model cannot generate a rapid and substantial viral decline after the peak unless a very large death rate of infected cells is chosen. However, only increasing the death rate of infected cells will decrease the first peak and eliminate the viral plateau/second peak, which is observed in all the 6 ponies. In this paper, we developed a new model (Eq. (1)) and showed that cytolysis of infected cells mediated by cytokines and cells such as NK cells during the innate immune response, can explain the rapid viral decline after peak.

During an early stage of infection, NK cell activity contributes to a rapid termination of many virus infections, including influenza, before the onset of the adaptive immune response [11], [47], [48], [49], [50]. Several studies in mice have illustrated that depletion of NK cells resulted in increased morbidity and mortality from influenza infection [51], [52], [53]. In humans, severe/lethal 2009 H1N1 influenza virus infection in 3 cases was associated with reduction of NK cells rather than effector CD8^+^ T cells [54], and influenza vaccination led to increased levels of NK cells with activation markers CD56 and CD69 [55]. NK cells are not only responsible for producing antiviral cytokines, but they are also directly involved in destroying virus-infected cells via the recognition by the natural cytotoxicity receptors (NCR) NKp46 (NCR1 in mice [6]) and NKp44 [7], [8], [9], [10]. Gazit et al. [6] showed that influenza virus infection was lethal in mice when the NK receptor NCR1 was knocked out.

In our model, we assumed that the level of activated NK cells is proportional to that of IFN, whose levels were measured in the study [16]. There is evidence supporting that NK cells have similar dynamics to IFN and virus during influenza virus infection. For example, an experimental study on murine influenza virus infection [56] showed that the effector cells with the properties of NK cells had very similar dynamics to the IFN level changes, i.e., peaked at 1–2 days post-infection and decreased to low levels by day 6. In mice that were inoculated intranasally with the mouse-adapted strain of human influenza A/PR/8/34 (H1N1) virus, the timing of viral peak and subsequent decline was consistent with that of NK cell-mediated cytolysis [57]. Another study [58] also showed that the peak of NK cells occurred within the first several days after influenza virus infection in mice, consistent with the timing of IFN production. In addition to the killing by IFN activated NK cells, high expression of cytokines during the innate immune response may also lead to infected cell death [34]. For example, influenza A virus-stimulated apoptosis was shown to be enhanced by IFN α/β and by increased expression of the antiviral protein PKR [35]. Macrophage-derived TRAIL (tumor necrosis factor-related apoptosis-inducing ligand) also plays an important role in promoting epithelial cell apoptosis [33].

We used IFN as a proxy of the innate immune response to model the cell-mediated lysis of infected epithelial cells and the antiviral effect. This may not be accurate because a number of other cytokines are involved in the innate immune response. Dendritic cells (DCs) and macrophages produce large amounts of antiviral and immunostimulatory cytokines in response to influenza virus infection [2], [4], [59], [60], [61]. We assumed that IFN is secreted by epithelial cells once they are infected. Other cells, such as monocytes, macrophages, and plasmacytoid DCs, can also contribute to IFN production [4], [37], [62]. Further, there may exist a time delay in IFN production, as observed in pony 1 (Figure 3) in which viral titer/infected cells peaked at day 2 post-infection while IFN peaked at day 3 post-infection. A similar time lag was observed in mice with influenza virus infection [63]. Moltedo et al. [63] showed that the initiation of lung inflammation (generation of IFNs, cytokines, chemokines, etc) did not begin until almost 2 days after infection, when virus replication reached its peak. This delay may be mediated by the influenza-encoded NS1 protein [63], which can act to block IFN production in influenza infected cells [48], [64], [65]. The burst of IFN production after day 2 might be explained by activation of plasmacytoid DCs or other uninfected cells in the lung, which are activated to a degree that correlates with viral titer or number of infected cells. Future comprehensive models may wish to take macrophages, DCs and other cytokines into account. However, more complicated models should be accompanied with appropriate data for model verification.

After the rapid post-peak decline of viral titer, we observed a plateau phase and/or the second viral peak. Although a number of models have been developed to study within-host influenza virus dynamics, very few models can generate the second peak. As the innate immune response weakens (Figure 3 shows that a rapid IFN decay was observed in all ponies even when the viral load was still high), the killing of infected cells (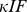) lapses in our model. Thus, the level of infected cells can remain unchanged for a while or even increase. This can explain the viral plateau and the second viral increase.

Another factor leading to the second peak is the augmented availability of target cells. The rapid IFN decay significantly reduces the conversion of susceptible cells to the refractory class. Because cells are most likely unable to maintain the antiviral state for a long time without continued IFN signaling, those cells that are already in the refractory class will revert back to the susceptible state and become the target of virus infection again. This will enhance the viral production. Some other factors may also contribute to the second peak. For example, when virus spreads to a previously uninvolved site in the lung or respiratory tract as discussed in [20], viral infection and production will increase and may lead to a second viral load increase.

After reaching the second peak around day 6 post-infection, the viral titer underwent a rapid second viral decline to below the detection limit. We showed that this second viral decline can be generated by the emergence of an adaptive immune response (Figure 2), which usually arises 4 to 7 days post-infection [11]. Without introducing an adaptive immune response in the model, the virus will not be cleared in ponies with a plateau/second peak. Because CD8^+^ T cell were not measured for these ponies, we assumed an increasing death rate of infected epithelial cells, δ_A_, after the second peak. We have also examined a model with an explicit adaptive immune response by adding another variable *X*, representing cytotoxic T lymphocytes (CTL), with *dX/dt = rX*, where *r* is the net expansion rate. We assumed the CTL-mediated killing of infected cells is *−kXI* in addition to *δ_I_I* in the model. In order for the adaptive immune response to remain at a very low level during the first several days, *r* should be very small. However, such a low-level adaptive immune response cannot generate the rapid second viral decline. This problem can be resolved by using a larger *r* and a time delay for the emergence of the adaptive immune response. However, this method is almost the same as what we did in the main text: increasing the death rate of infected cells several days after infection.

In addition to CD8^+^ T cells, antibodies neutralizing free virions may also be involved in viral clearance. Increasing either the infected cell death rate *δ*, as shown in our study, or the viral clearance rate *c* can generate the same second viral decline to below the detection limit. Thus, from the comparison between model predictions and the data, we cannot determine if the viral clearance is mainly caused by CD8^+^ T cells or neutralizing antibodies. However, in the experiment [16] from which we studied the data, no anti-influenza antibodies were detected by the SRH assay 7 days post-challenge in any of the ponies. Low levels of antibodies were detected by ELISA on day 7 for 3 of the 6 ponies. Although such antibodies may exist at low levels before day 7, they may not be the major factor responsible for viral clearance because the infection was already resolved by day 7 in ponies 5 and 6. Likewise, we cannot estimate the duration of the eclipse phase in which infected cells have not started to produce virions because the model with and without an eclipse phase both fit the experimental data well (Supporting Text S1, Table S1, Table S2, Figures S9, and S10).

Although target cells are not depleted, we predict a decline of target cells as well as the total number of epithelial cells during infection (Figure 4). The reason for the decline is that we did not include generation/proliferation of epithelial cells. This is not important for the short time period of infection we studied. Consistent with the other studies [19], [20], [21], including the regeneration of target epithelial cells in our model does not improve the fits of the model to the data set. This is also supported by the observation in humans that regenerating respiratory epithelium cells appeared only in 3 out of 14 subjects after 5–14 days post-infection [66], whereas virus infection is usually resolved within 7–10 days [67]. Once the virus is cleared, generation/proliferation will increase epithelial cells to the pre-infection level.

In summary, by fitting mathematical models to the viral load and IFN data we illustrate that both the innate and adaptive immune responses are needed to explain the viral load change during influenza virus infection. The first post-peak viral decline (about 2 to 4 logs within 1 day) can be explained by the lysis of infected epithelial cells, mediated by cytokines and cells such as NK cells, during the innate immune response. The subsequent viral plateau/second peak is generated in our model by the loss of the IFN-induced antiviral effect and the increased availability of target cells as cells lose their antiviral state. An adaptive immune response is needed in our model to explain the eventual viral clearance. A detailed and quantitative study of the within-host dynamics of virus, cells, and cytokines may provide more information for future research in influenza pathogenesis, treatment, and vaccination.

## Supporting Information

Figure S1
**Best fits of different models to the IFN data.** Model 1 is described by Eq. (1). Model 2 is Eq. (1) with κ = 0, i.e., no killing of infected cells by NK cells. Model 3 is model 2 assuming the viral production rate is 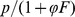.(TIFF)

Figure S2 **Sensitivity tests of predicted viral load to parameters.** The first four rows: sensitivity tests of the predicted viral load of pony 1 to model parameters (Eq. (1)). The parameter in the legend was varied while the remaining parameters were fixed and chosen from Table 2. The fifth row: best fits of Eq. (1) assuming the initial number of target cells is 75% or 50% of 3.5×10^11^ cells to the viral load data. The best-fit parameters are shown in Table S3.
(TIF)

Figure S3 **Sensitivity tests of predicted interferon level to parameters.** The parameter in the legend was varied while the remaining parameters were fixed and chosen from Table 2.
(TIF)

Figure S4 **Contour plots of the viral load as a function of the indicated parameter and time.** On the right side of each contour plot there is a color scale in which different colors represent different viral loads (in the log scale).
(TIF)

Figure S5 **Contour plots of interferon as a function of the indicated parameters and time.**
(TIF)

Figure S6 **Contour plots of the viral load peak as a function of the indicated parameters.**
(TIF)

Figure S7 **Contour plots of the interferon peak as a function of the indicated parameters.**
(TIF)

Figure S8 **Contour plots of the viral load as a function of the indicated parameters and time.**
(TIF)

Figure S9 **Best fits of the eclipse model to the viral load data.** The horizontal dashed blue line represents the detection limit of the viral titer, i.e., 100 RNA copies per ml of nasal secretions. Data below the detection limit were plotted as 1 RNA copy per ml of nasal secretions.
(TIF)

Figure S10 **Best fits of the eclipse model to the IFN data.**
(TIF)

Table S1 **Parameter values of the best fits of the eclipse model to experimental data.**
(PDF)

Table S2 **Comparisons of the best fits using different models.**
(PDF)

Table S3 **Parameter values of the best fits of** **Eq. (1)** **with reduced T_0_ to the data of pony 1.**
(PDF)

Table S4 **Comparisons of the best fits using AIC.**
(PDF)

Text S1 **The model with an eclipse phase.**
(PDF)
