# Supplementary figures and images for: Modeling Within-Host Dynamics of Influenza Virus Infection Including Immune Responses
Source: PLoS Comput Biol. 2012 Jun 28;8(6):e1002588. doi: 10.1371/journal.pcbi.1002588 (PMC3386161; doi:10.1371/journal.pcbi.1002588)

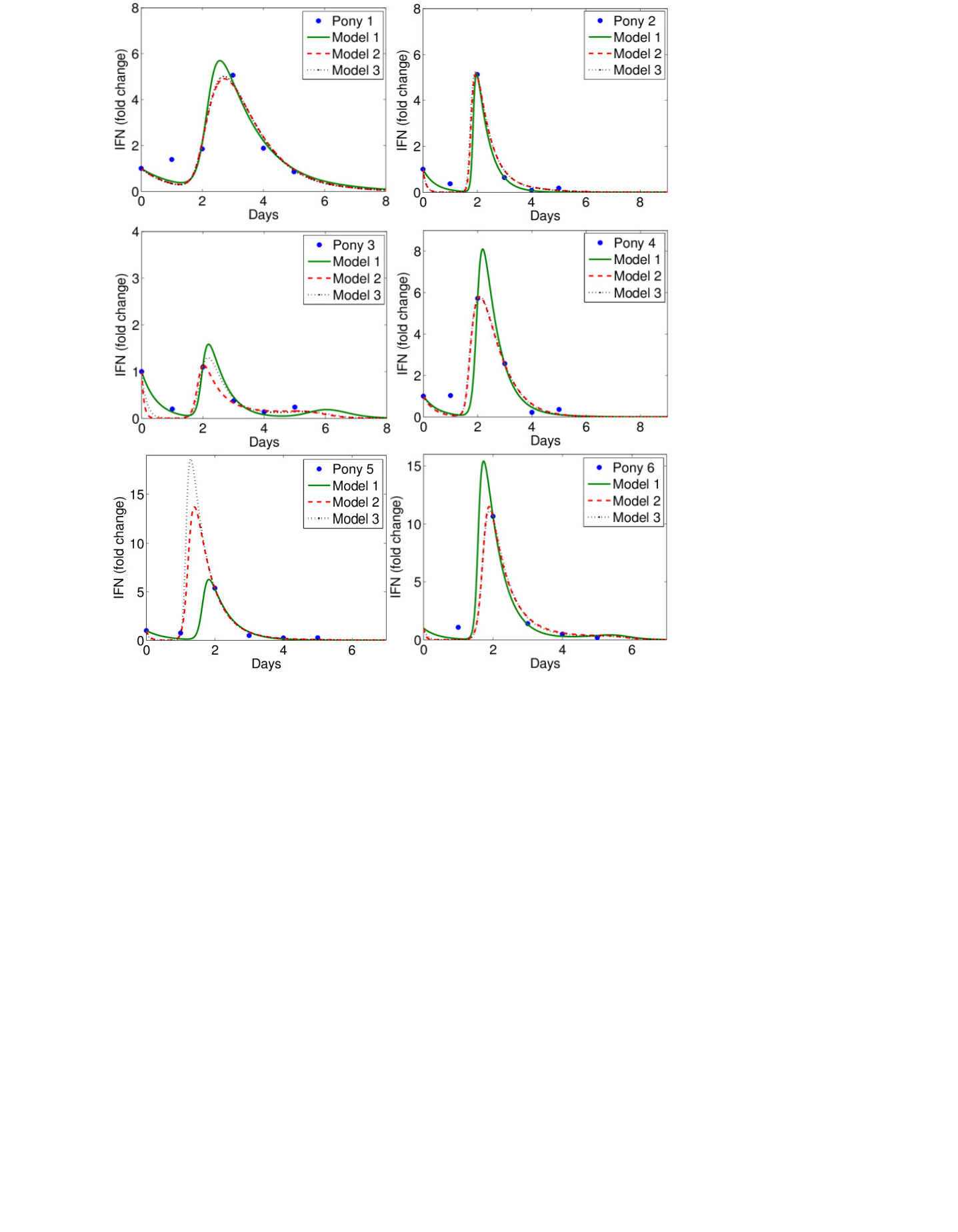

Supplement: Figure S1 — Best fits of different models to the IFN data. Model 1 is described by Eq. (1). Model 2 is Eq. (1) with κ = 0, i.e., no killing of infected cells by NK cells. Model 3 is model 2 assuming the viral production rate is . (TIFF) [file pcbi.1002588.s001.tif]

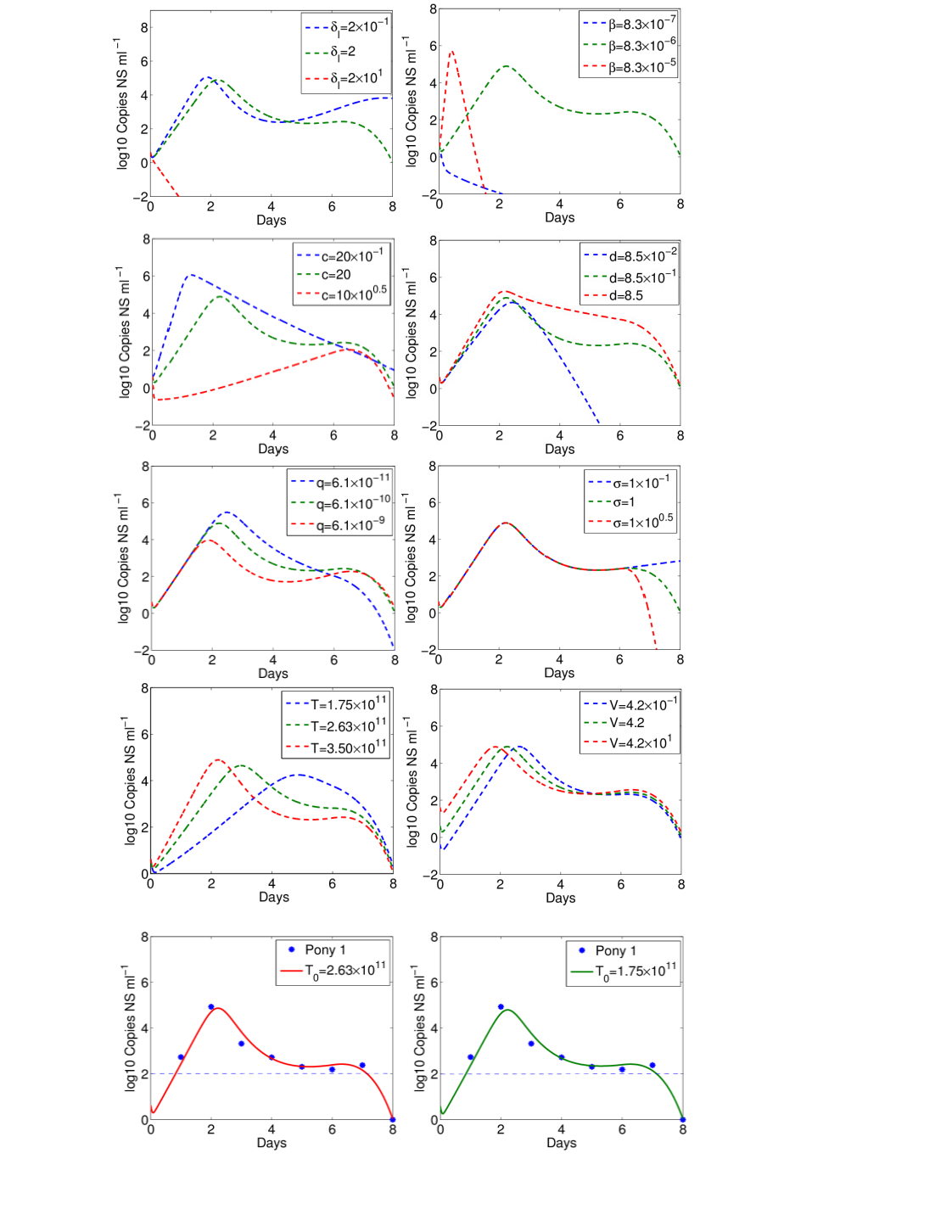

Supplement: Figure S2 — Sensitivity tests of predicted viral load to parameters. The first four rows: sensitivity tests of the predicted viral load of pony 1 to model parameters (Eq. (1)). The parameter in the legend was varied while the remaining parameters were fixed and chosen from Table 2. The fifth row: best fits of Eq. (1) assuming the initial number of target cells is 75% or 50% of 3.5×10^11^ cells to the viral load data. The best-fit parameters are shown in Table S3. (TIF) [file pcbi.1002588.s002.tif]

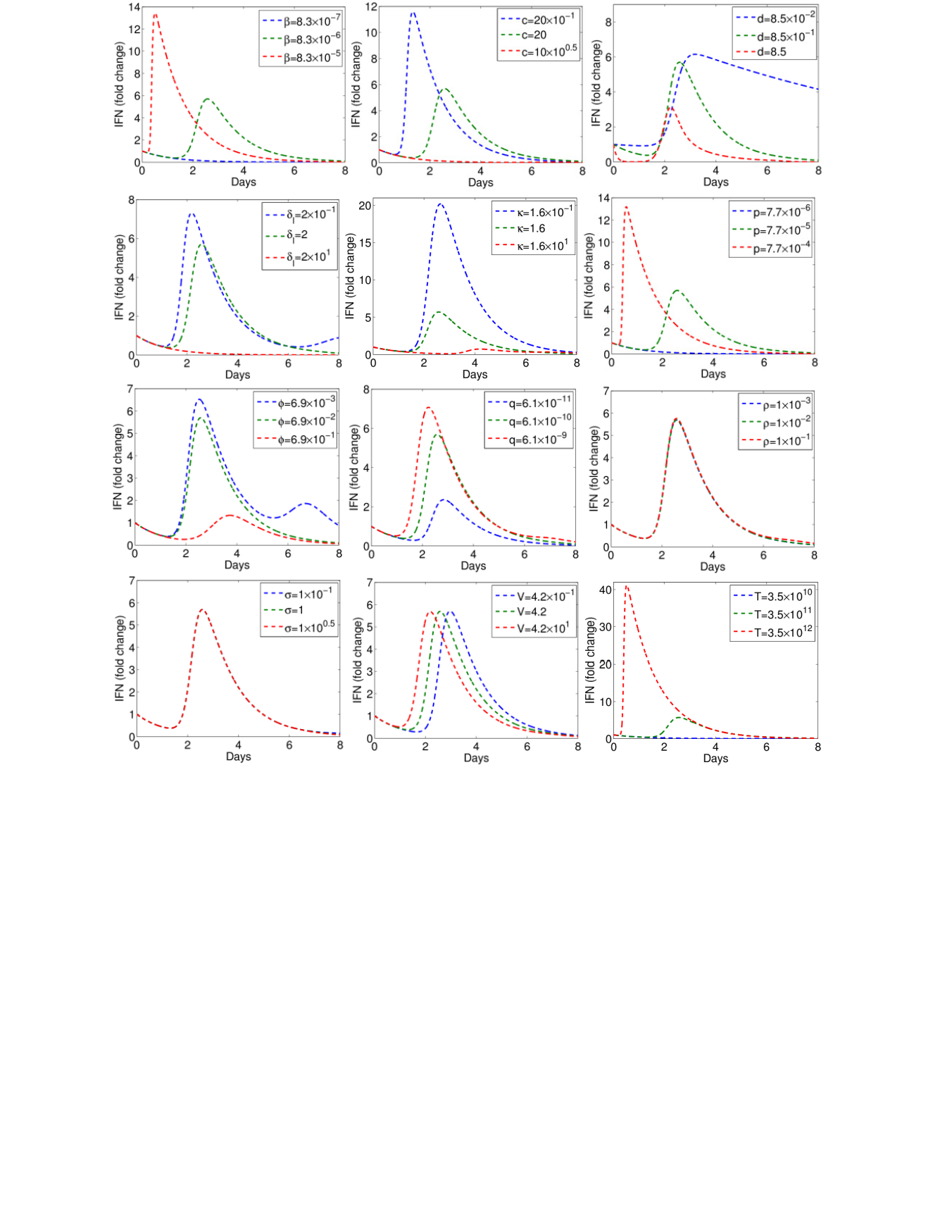

Supplement: Figure S3 — Sensitivity tests of predicted interferon level to parameters. The parameter in the legend was varied while the remaining parameters were fixed and chosen from Table 2. (TIF) [file pcbi.1002588.s003.tif]

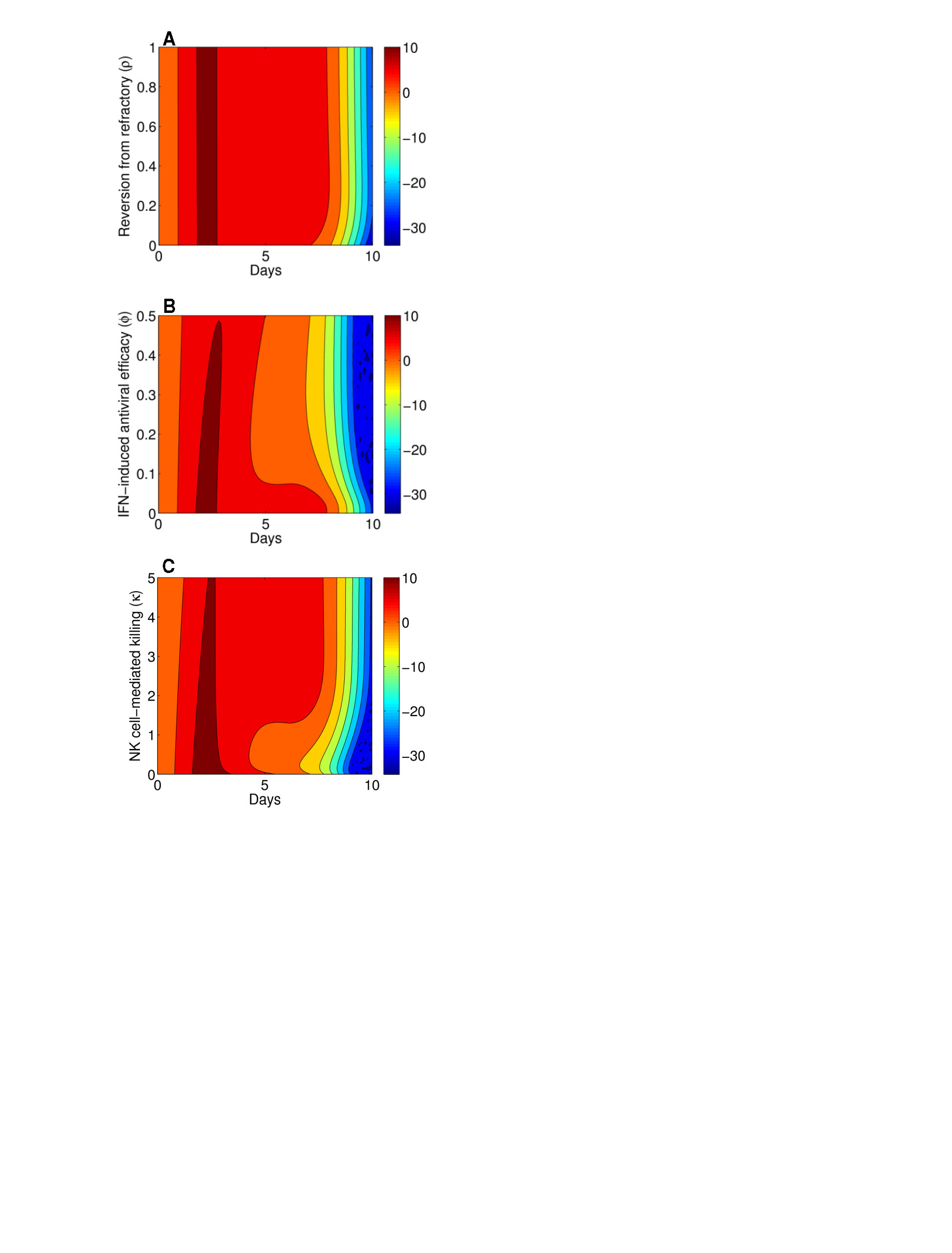

Supplement: Figure S4 — Contour plots of the viral load as a function of the indicated parameter and time. On the right side of each contour plot there is a color scale in which different colors represent different viral loads (in the log scale). (TIF) [file pcbi.1002588.s004.tif]

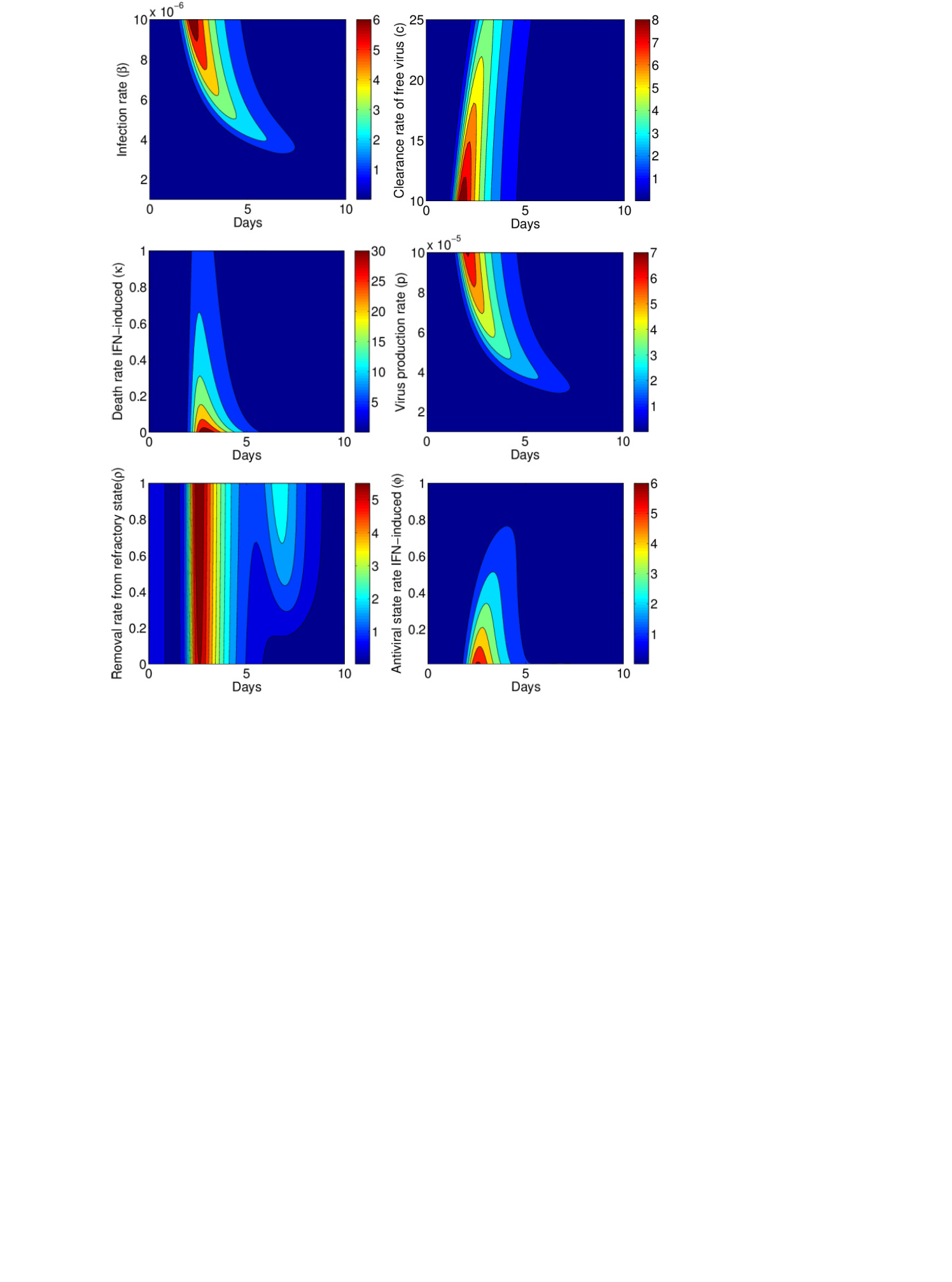

Supplement: Figure S5 — Contour plots of interferon as a function of the indicated parameters and time. (TIF) [file pcbi.1002588.s005.tif]

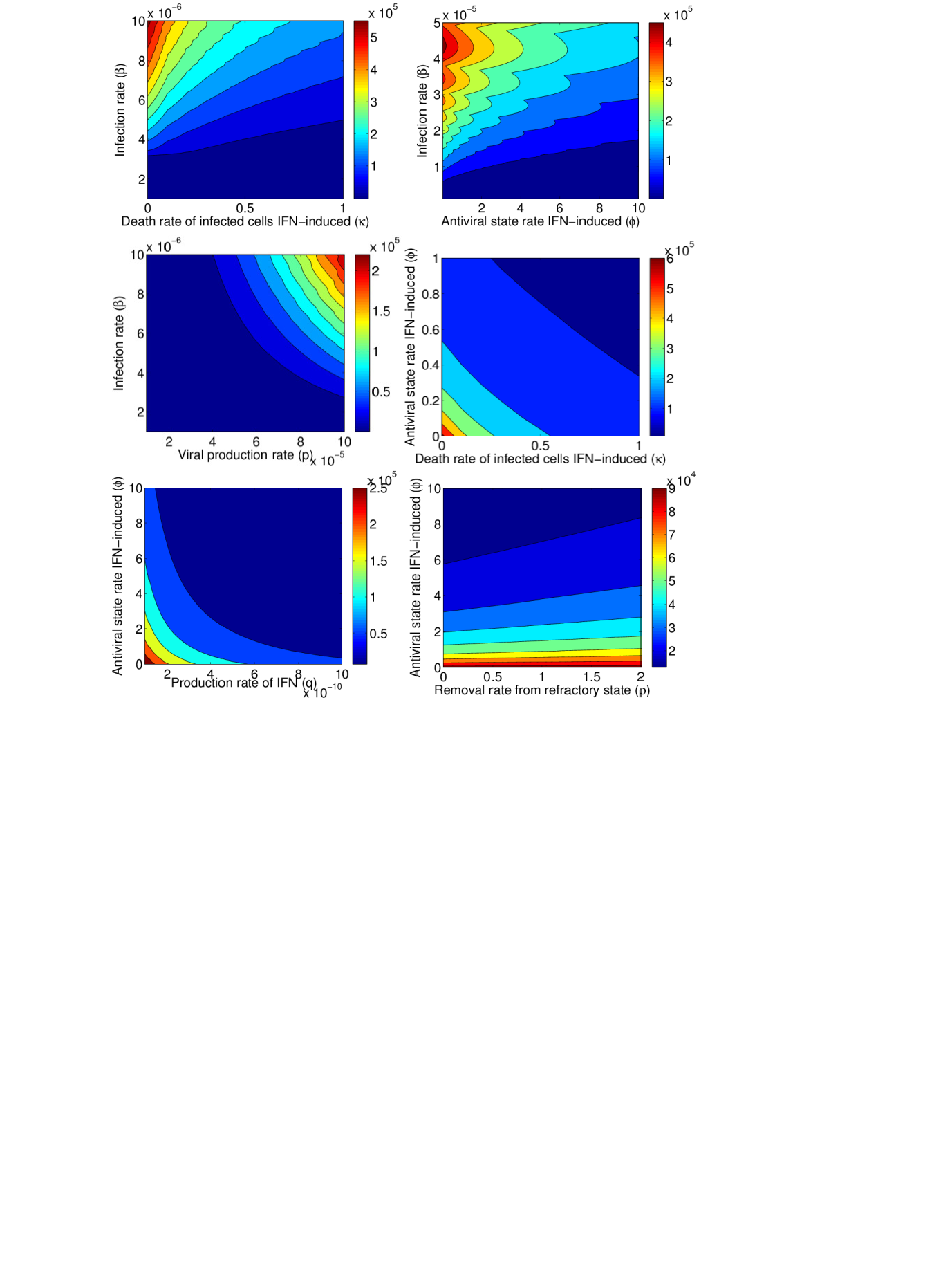

Supplement: Figure S6 — Contour plots of the viral load peak as a function of the indicated parameters. (TIF) [file pcbi.1002588.s006.tif]

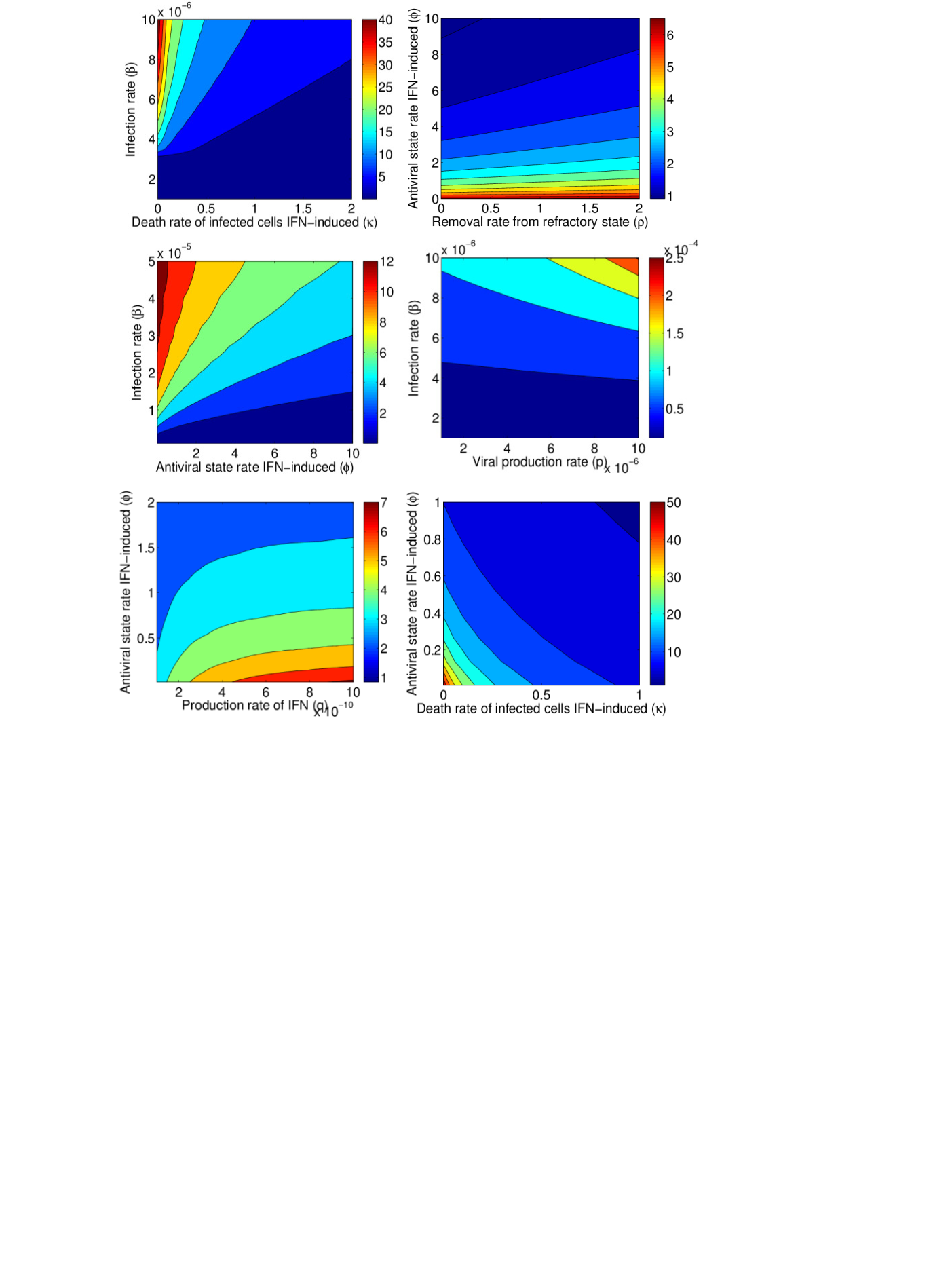

Supplement: Figure S7 — Contour plots of the interferon peak as a function of the indicated parameters. (TIF) [file pcbi.1002588.s007.tif]

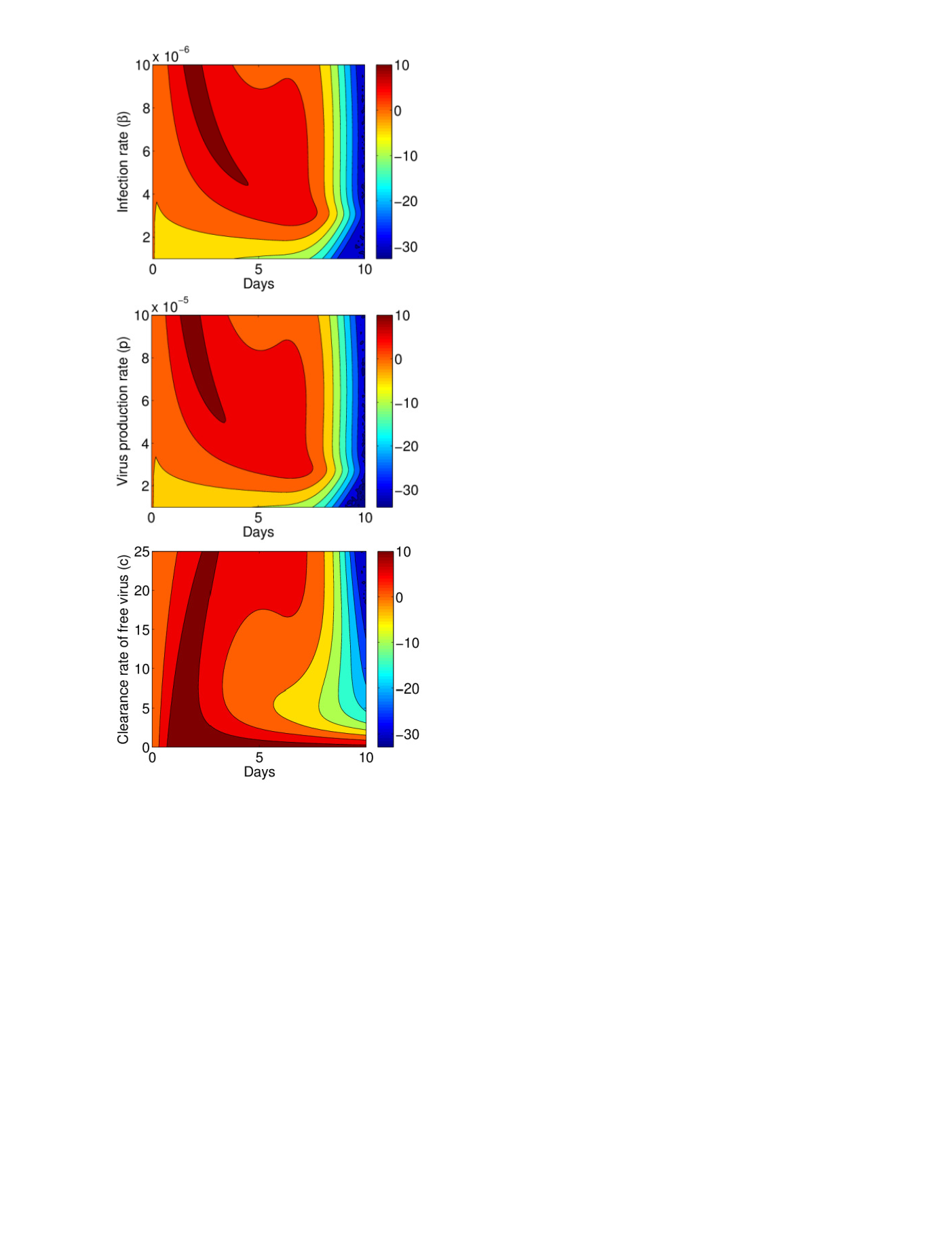

Supplement: Figure S8 — Contour plots of the viral load as a function of the indicated parameters and time. (TIF) [file pcbi.1002588.s008.tif]

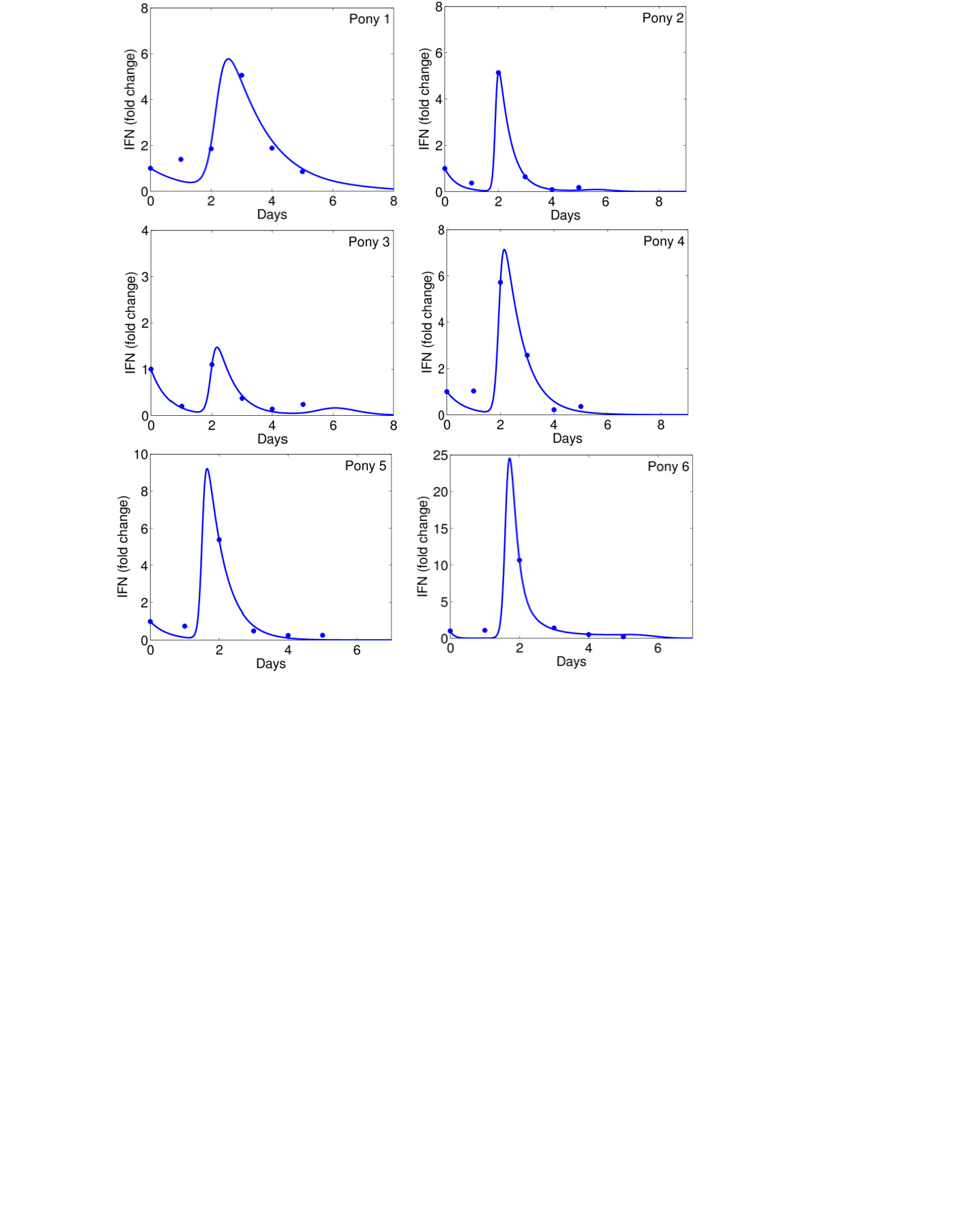

Supplement: Figure S10 — Best fits of the eclipse model to the IFN data. (TIF) [file pcbi.1002588.s010.tif]
