# Supplementary material for: Modeling Within-Host Dynamics of Influenza Virus Infection Including Immune Responses
Source: PLoS Comput Biol. 2012 Jun 28;8(6):e1002588. doi: 10.1371/journal.pcbi.1002588 (PMC3386161; doi:10.1371/journal.pcbi.1002588)
Supplement: Table S1 — Parameter values of the best fits of the eclipse model to experimental data. (PDF) [file pcbi.1002588.s011.pdf]

Table S1: Parameter values of the best fits of the model with an eclipse phase (Eq. (3)) to experimental data.

Pony	β	ϕ	ρ	κ	p	c	q	d	σ
	(RNA copy)$^{-1}$ ml NS day^{-1}	(IFN fold change)$^{-1}$ day^{-1}	day^{-1}	(IFN fold change)$^{-1}$ day^{-1}	RNA copies (ml NS)$^{-1}$ day^{-1} cell^{-1}	day^{-1}	IFN fold change day^{-1} cell^{-1}	day^{-1}	
1	1.3×10^{-3}	1.3×10^{-1}	5.8×10^{-3}	1.5	1.1×10^{-4}	20	7.7×10^{-9}	0.83	0.95
2	1.8×10^{-6}	1.1×10^{0}	4.1×10^{-3}	4.7	6.9×10^{-3}	4.2	2.9×10^{-9}	2.3	1.4
3	2.7×10^{-4}	5.3×10^{-1}	8.4×10^{-2}	8.5	1.4×10^{-5}	10	2.1×10^{-10}	1.8	0.75
4	1.3×10^{-6}	1.0×10^{-1}	1.5×10^{1}	2.3	2.2×10^{-3}	7.1	1.1×10^{-9}	1.5	0.30
5	1.4×10^{-6}	1.3×10^{0}	3.5×10^{-3}	2.1	9.5×10^{-3}	9.0	4.9×10^{-9}	2.0	1.3
6	5.6×10^{-4}	1.4×10^{0}	8.3×10^{-3}	0.33	1.8×10^{-5}	14	1.4×10^{-8}	9.0	1.0
Average	3.6×10^{-4}	7.6×10^{-1}	2.5×10^{0}	3.2	3.1×10^{-3}	11	5.1×10^{-9}	2.9	0.95
