# Supplementary material for: Modeling Within-Host Dynamics of Influenza Virus Infection Including Immune Responses
Source: PLoS Comput Biol. 2012 Jun 28;8(6):e1002588. doi: 10.1371/journal.pcbi.1002588 (PMC3386161; doi:10.1371/journal.pcbi.1002588)
Supplement: Table S2 — Comparisons of the best fits using different models. (PDF) [file pcbi.1002588.s012.pdf]

Table S2: Comparisons of the best fits using different models.

Pony	RMS Model 1*	AICc**	RMS The eclipse model*	AICc**
1	0.695	-10.701	0.690	-10.774
2	0.651	-11.740	0.726	-10.540
3	0.320	-19.069	0.319	-19.105
4	0.768	-9.932	0.811	-9.351
5	0.618	-11.486	0.597	-11.816
6	0.695	-10.385	0.720	-10.060

* Model 1 is described by Eq. (1). The eclipse model (model 1 with an eclipse phase) is given by Eq. (3).

** Calculated by Eq. (4).
