# Supplementary material for: Modeling Within-Host Dynamics of Influenza Virus Infection Including Immune Responses
Source: PLoS Comput Biol. 2012 Jun 28;8(6):e1002588. doi: 10.1371/journal.pcbi.1002588 (PMC3386161; doi:10.1371/journal.pcbi.1002588)
Supplement: Table S3 — Parameter values of the best fits of Eq. (1) with reduced T0 to the data of pony 1. (PDF) [file pcbi.1002588.s013.pdf]

Table S3: **Parameter values of the best fits of Eq. (1) with reduced initial numbers of target cells (T_0) to the data of pony 1.**

T_0	β	ϕ	ρ	κ	p	c	q	d	σ
	(RNA copy)$^{-1}$ ml NS day^{-1}	(IFN fold change)$^{-1}$ day^{-1}	day^{-1}	(IFN fold change)$^{-1}$ day^{-1}	RNA copies (ml NS)$^{-1}$ day^{-1} cell^{-1}	day^{-1}	IFN fold change day^{-1} cell^{-1}	day^{-1}	
2.6×10^{11}	8.6×10^{-6}	6.9×10^{-2}	1.0×10^{-2}	1.6	1.0×10^{-4}	20	8.1×10^{-10}	0.85	1.0
1.8×10^{11}	2.2×10^{-5}	2.6×10^{-2}	6.7×10^{-2}	1.2	5.5×10^{-5}	20	5.4×10^{-10}	0.85	1.0
