# Supplementary material for: Modeling Within-Host Dynamics of Influenza Virus Infection Including Immune Responses
Source: PLoS Comput Biol. 2012 Jun 28;8(6):e1002588. doi: 10.1371/journal.pcbi.1002588 (PMC3386161; doi:10.1371/journal.pcbi.1002588)
Supplement: Table S4 — Comparisons of the best fits using AIC. (PDF) [file pcbi.1002588.s014.pdf]

Table S4: Comparisons of the best fits using AIC.

Pony	RMS Model 1*	AICc**	RMS model 2*	AICc**	Number of data points***
1	0.695	-10.701	0.951	-7.785	14
2	0.651	-11.740	1.061	-6.762	15
3	0.320	-19.069	0.718	-10.471	14
4	0.768	-9.932	0.953	-7.808	15
5	0.618	-11.486	1.027	-7.121	13
6	0.695	-10.385	0.845	-8.738	13

* Model 1 is described by Eq. (1). Model 2 is Eq. (1) with κ=0, i.e., there is no killing of infected cells by NK cells.
** Calculated by Eq. (4).
*** We did not include data points of viral titer under the detection limit after the first undetectable data point.
