# Supplementary material for: Modeling Within-Host Dynamics of Influenza Virus Infection Including Immune Responses
Source: PLoS Comput Biol. 2012 Jun 28;8(6):e1002588. doi: 10.1371/journal.pcbi.1002588 (PMC3386161; doi:10.1371/journal.pcbi.1002588)
Supplement: Text S1 — The model with an eclipse phase. (PDF) [file pcbi.1002588.s015.pdf]

Supporting Text S1

The model with an eclipse phase. It was observed that virus particles did not appear until 6-8 hours after the virus enters an uninfected cell [1]. Another experimental study showed that new virus particles were detectable 4-12 hours post infection [2]. To account for this delay in the viral production an eclipse phase is introduced by separating infected cells into two categories, namely, infected cells not yet producing virus particles (I_1) and virus-producing cells (I_2). We assume that after an average time of $1/k$ since viral entry the cell starts to release virus. This feature was also included in other mathematical models, for example, in [3] and [4]. Note that we did not include the death rate of infected cells in the equation of I_1 because the eclipse phase is much shorter than the lifespan of an infected cell. Similar to previous modeling studies [3,4], we assume that IFN is secreted only by productively infected cells (I_2). The other parameters including the time-varying death rate of infected cells after the emergence of an adaptive immune response have the same definitions as in Eq. (1) in the main text. The model is given by the following system of equations:

$$
\begin{aligned}
\frac{dT}{dt} &= -\beta VT - \phi FT + \rho R \\
\frac{dI_1}{dt} &= \beta VT - kI_1 - \kappa I_1 F \\
\frac{dI_2}{dt} &= kI_1 - \delta I_2 - \kappa I_2 F \\
\frac{dR}{dt} &= \phi FT - \rho R \\
\frac{dV}{dt} &= pI_2 - cV \\
\frac{dF}{dt} &= qI_2 - dF
\end{aligned}
\qquad (3)
$$

To decide if the eclipse model can explain the viral kinetics described in the main text, we fit it to the same experimental data (Figure S9 for viral load and Figure S10 for IFN fold change). While performing data fitting we set k to 4 day^{-1}, which is the best fit estimate from [3] and was also used in [4]. Equation (3) with a fixed parameter k has the same number of parameters as Eq. (1).

Through data fitting, we obtained parameters estimates for the eclipse model (Table S1). We also calculated the error between modeling predictions and experimental data (RMS). These values for model 1 (Eq. (1)) and the eclipse model (Eq. (3)) are given in Table S2. They are similar for the two models.

In order to statistically compare the best fits of using model 1 and the eclipse model, we calculated the modified Akaike Information Criterion (AICc) [5]. The model with a lower AICc value fits the data better from a statistical viewpoint. We used the following formula to calculate the AICc:

$$AICc = 2(m+1) + n\log\left(RMS\right)^2 + \frac{2(m+1)(m+2)}{n-m}, \quad (4)$$

where m is the number of fitted parameters and n is the number of data points [5]. The RMS value is the root of the mean squared residual. Comparison between models was performed individually for all ponies. The AICc values are given in Table S2. Model 1 provides a slightly better fit for some ponies. However, the difference is not significant. This suggests that the model with and without an eclipse phase both fit the data well.

REFERENCES

1. Sedmak JJ, Grossberg SE (1973) Interferon bioassay: reduction in yield of myxovirus neuraminidases. J Gen Virol 21: 1-7.

2. Rimmelzwaan GF, Baars M, Claas EC, Osterhaus AD (1998) Comparison of RNA hybridization, hemagglutination assay, titration of infectious virus and immunofluorescence as methods for monitoring influenza virus replication in vitro. J Virol Methods 74: 57-66.

3. Baccam P, Beauchemin C, Macken CA, Hayden FG, Perelson AS (2006) Kinetics of influenza A virus infection in humans. J Virol 80: 7590-7599.

4. Handel A, Longini IM, Jr., Antia R (2010) Towards a quantitative understanding of the within-host dynamics of influenza A infections. J R Soc Interface 7: 35-47.

5. Burnham K, Anderson D (2002) Model selection and multimodel inference: New York, Springer.
